# Invagination of Ectodermal Placodes Is Driven by Cell Intercalation-Mediated Contraction of the Suprabasal Tissue Canopy

**DOI:** 10.1371/journal.pbio.1002405

**Published:** 2016-03-09

**Authors:** Eleni Panousopoulou, Jeremy B. A. Green

**Affiliations:** Department of Craniofacial Development & Stem Cell Biology, King’s College London, London, United Kingdom; Stanford University School of Medicine, Howard Hughes Medical Institute, UNITED STATES

## Abstract

Ectodermal organs such as teeth, hair follicles, and mammary glands begin their development as placodes. These are local epithelial thickenings that invaginate into mesenchymal space. There is currently little mechanistic understanding of the cellular processes driving the early morphogenesis of these organs and of why they lead to invagination rather than simple tissue thickening. Here, we show that placode invagination depends on horizontal contraction of superficial layers of cells that form a shrinking and thickening canopy over underlying epithelial cells. This contraction occurs by cell intercalation and is mechanically coupled to the basal layer by peripheral basal cells that extend apically and centripetally while remaining attached to the basal lamina. This process is topologically analogous to well-studied apical constriction mechanisms, but very different from them both in scale and molecular mechanism. Mechanical cell–cell coupling is propagated through the tissue via E-cadherin junctions, which in turn depend on tissue-wide tension. We further present evidence that this mechanism is conserved among different ectodermal organs and is, therefore, a novel and fundamental morphogenetic motif widespread in embryonic development.

## Abstract 2

A study of teeth, hair follicles, and mammary ducts reveals that the superficial layer of the initial tissue thickening, or placode, contracts by a novel form of cell intercalation. This exerts a bending force to pinch the underlying layer into a fold.

## Author Summary

Teeth, hair follicles, and skin ducts (including mammary and sweat glands) are initially formed in the embryo as slight thickenings of a flat epithelium that are called placodes. These then invaginate to form dimples or pits that make the characteristic structures found in the adult. While some invagination mechanisms are well-studied and it is recognized that invagination is one of the basic motifs needed to construct the body, the physical events that lead placodes to invaginate are unclear. Here, we analyzed the events required to form tooth placodes and identified a novel mechanism: we showed that the superficial layer of the placode contracts to pucker the underlying epithelium, ultimately forcing it deep into the underlying mesenchyme. We demonstrated that the superficial tissue generates contractile forces and that the mechanical tension deforms nuclei in this tissue. This allowed us to map the tension not only in developing teeth, but also in hair follicles and mammary glands, revealing similar patterns of nuclear distortion in different tissues and suggesting the existence of a shared mechanism of invagination. We also labelled individual cells and tracked them in real time, showing that the tissue contracts via cell intercalation, with some cells remaining anchored to the basal layer of the epithelium while trying to migrate toward the placode centre. Overall, our results describe the dynamic rearrangements that take place during tooth placode formation and suggest that similar processes occur in other organs that are formed by invagination of stratified placodes.

## Introduction

**Fig 1:**
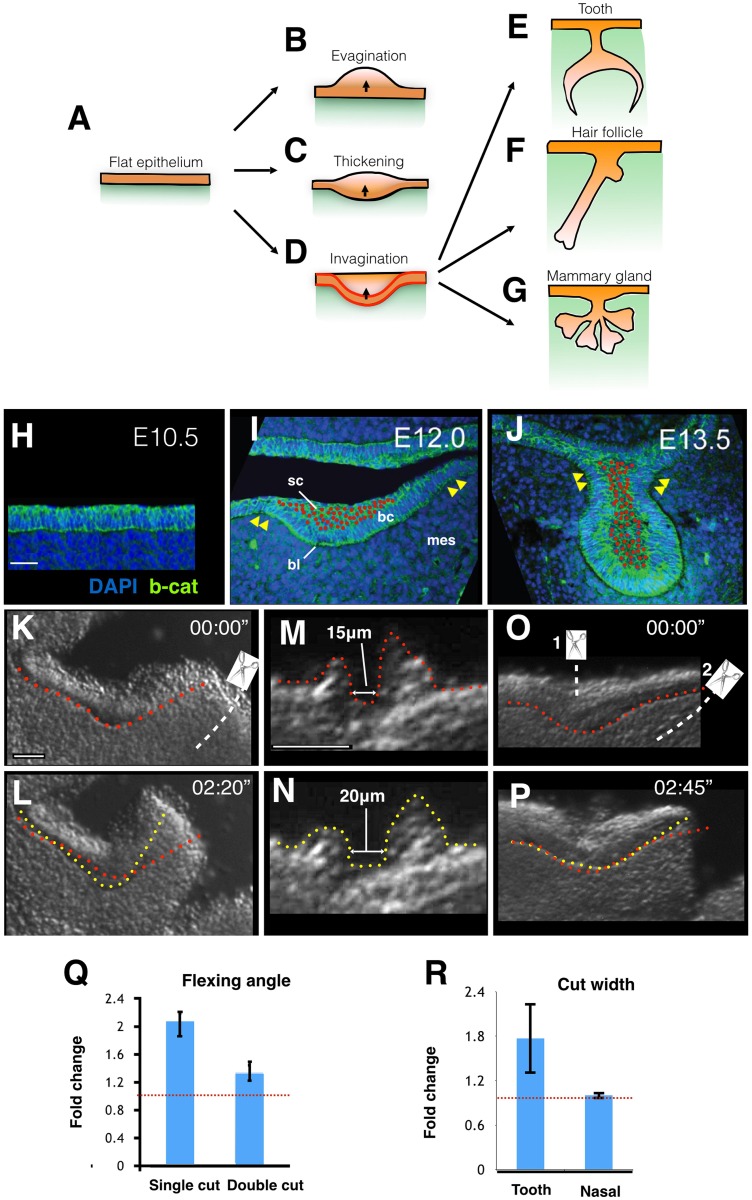
Contractile suprabasal tissue drives bending of the tooth placode. **A–G.** Epithelial invagination is an early and critical step in the development of ectodermal organs like the tooth, hair follicle, and mammary gland. Locally increased proliferation or vertically orientated cell divisions (vertical arrows in B–D), which are textbook models of tooth invagination, do not explain why the resulting structure should invaginate (D) versus thicken (C) or evaginate (B). I.e., local stratification alone cannot explain the observed morphogenesis, specifically, why the basal profile bends to become concave. Active invagination of the basal cell layer (outlined red in D) is one hypothesis. **H–J.** Frontal views of the indicated stages of embryonic molar tooth development. Red dots mark all suprabasal cells; arrowheads indicate shoulders; bl = basal lamina, bc = basal cells, sc = suprabasal cells, mes = mesenchyme. **K–P.** Video frames (S2 and S3 Movies) showing tissue outline (M,N) or basal lamina shape (K,L,O,P) immediately upon cutting (red lines) or at a later indicated time (yellow lines). **K,L.** Tooth placode rapid bending upon lateral cut. **M,N.** Suprabasal cut (close-up of the cut; full view can be found in S3 Movie) shows rapid increase in width. **O,P.** Suprabasal cut (cut 1) disables lateral cut- (cut 2) induced bending. **Q.** Bending angles of indicated experiments (mean +/- SD [standard deviation]). **R.** Recoils from tooth (*n* = 4) and nasal (*n* = 2) epithelium (mean +/- SD) (images of nasal epithelium are shown in S2A and S2B Fig). All scale bars are 30 μm.

Understanding how tissues form physically (morphogenesis) is a major frontier, both in developmental biology and organ regeneration using stem cells [1,2]. Epithelial bending, especially invagination, is a recurrent morphogenetic event in development [3–12], but our knowledge of the underlying cellular mechanisms is quite limited. Ectodermal organs, such as teeth, hair follicles, and mammary glands all depend on epithelial bending at the start of their development: local epithelial thickenings (placodes) must invaginate into mesenchymal space for proper organ shaping (Fig 1A–1G) [13]. While molecular signaling involved in placode inductions is well described and well conserved [14,15], less attention has been given to the physical events required to execute this programme. The current view is that placodes thicken and invaginate either through vertically orientated cell divisions, in the case of teeth [16], and/or by centripetal cell migration, in the case of hair follicles [17]. However, neither of these processes as such can explain why the epithelium invaginates (Fig 1D) rather than causing cells to pile up, merely thickening the epithelium (Fig 1B and 1C).

One plausible hypothesis is that cell shape changes in the basal cell layer (cells in contact with the basal lamina) drive invagination (Fig 1D). Such monolayer invagination occurs in other well-studied contexts either by apical constriction, in which actinomyosin contractile fibers narrow the apical ends of cells, making them wedge- or cone-shaped [5,7,9–11], or “basal wedging” (observed in vertebrate neural tube formation), in which nuclei move basally in a pseudostratified epithelium to expand the basal area of highly columnar cells (S1A and S1B Fig) [3,10]. We set out to determine whether these processes or others might be the mechanism of placode invagination. We found that a third, entirely novel mechanism—suprabasal cell intercalation—is responsible.

## Results

To determine whether apical constriction or basal wedging contributes to placodal invagination, we analysed cells in mouse molar tooth primordia (Fig 1H–1J). We labeled cells mosaically using tamoxifen-inducible membrane-GFP and quantified apical and basal widths of basal layer cells in invaginated versus flanking flat epithelium (S1C–S1I Fig). Cells with basal nuclei were not more abundant, nor did they have significantly wider bases, in the invaginating epithelium versus adjacent flat epithelium (S1D and S1E Fig), ruling out the basal wedging mechanism. These cells did exhibit apical narrowing (S1E and S1F Fig; note that ratio of means is not equal to the mean of ratios) but, surprisingly, had no apical enrichment of actin filaments or activated (phospho-) myosin (pMLC) (S1G–S1I Fig), the hallmarks of apical constriction [5,7,9–11]. Instead, pMLC appeared elevated in horizontally elongated suprabasal cells (S1G Fig), as compared to underlying and neighbouring basal cells, suggesting that the superficial layers of the placode may be under contractile tension.

Suprabasal tissue in the tooth placode undergoes gross narrowing and deepening during invagination (red dots in Fig 1I and 1J), bringing together “shoulders” on either side of the invagination (yellow arrowheads in Fig 1I and 1J) to make a tooth bud with a narrow neck (Fig 1J). Tissue narrowing and lengthening, if active and autonomous, is termed convergent extension (CE), a highly conserved morphogenetic movement that simultaneously produces extending and contractile forces through coordinated cell rearrangements and drives processes ranging from early embryo axial extension to organ tubule extension [5,18–20]. To test whether suprabasal CE drives placode invagination, we first determined whether the invagination of the epithelium is mechanically independent of underlying mesenchyme by explanting it. Explants rapidly bent themselves in the direction of normal invagination (S1 Movie), showing that mesenchyme is not a mechanical requirement for epithelial bending. A partial excision (“lateral cut”) was sufficient to show that a 40° bend could take place in as little as 150 s (compared to 12 h in vivo) (Fig 1K and 1L; S2 Movie), indicating that bending is normally resisted by the attachment to flanking epithelium and underlying mesenchyme. The lateral cut, by releasing this resistance, allowed rapid elastic recoil from the incision site. Quantification showed the angle made by the epithelium after bending to be significantly less than the angle before cutting (*p*-value < 0.001, *t* test; Fig 1Q). Similar explants of nasal epithelium (a nearby noninvaginating pseudostratified epithelium) at E12.5 showed no bending behaviour. Manual re-extension and release of the bent epithelium graphically demonstrated its elastic nature (S1 Movie).

To determine whether the contractile forces driving bending are generated in the suprabasal layers specifically, we made fine incisions through these layers, leaving basal cells intact (Fig 1M and 1N; S2G–S2I Fig). We found that incisions immediately expanded by bidirectional recoil from 17 +/- 2 μm wide at the start of recording to 27 +/- 4 μm in less than one min (Fig 1M, 1N and 1R; S3 Movie). Similar incisions in nasal epithelium from littermate embryos did not recoil upon cutting (Figs 1R, S2A and S2B; S3 Movie). This confirmed that tensile forces in the plane are not a general property of thickened epithelium. After incubation for a further 4 min, cut explants gradually adopted a “W” shape as the basal epithelium flopped open below the incision (S2C–S2F Fig), showing that the intact basal layer below the cut was unable to sustain the bend at this position. To test directly the insufficiency of the basal layer and requirement for suprabasal tissue, we released suprabasal tension with a suprabasal cut and then followed it by a lateral cut. If the suprabasal contractile force is required for invagination, then releasing it should result in loss of lateral cut-induced bending. This is what we observed, with no significant change in epithelial bend (*p*-value = 0.11; *t* test; Fig 1O–1Q; S2 Movie). Thus, intact, contractile suprabasal tissue is required for bending, providing force for bending-associated apical narrowing of basal layer cells extrinsically.

**Fig 2:**
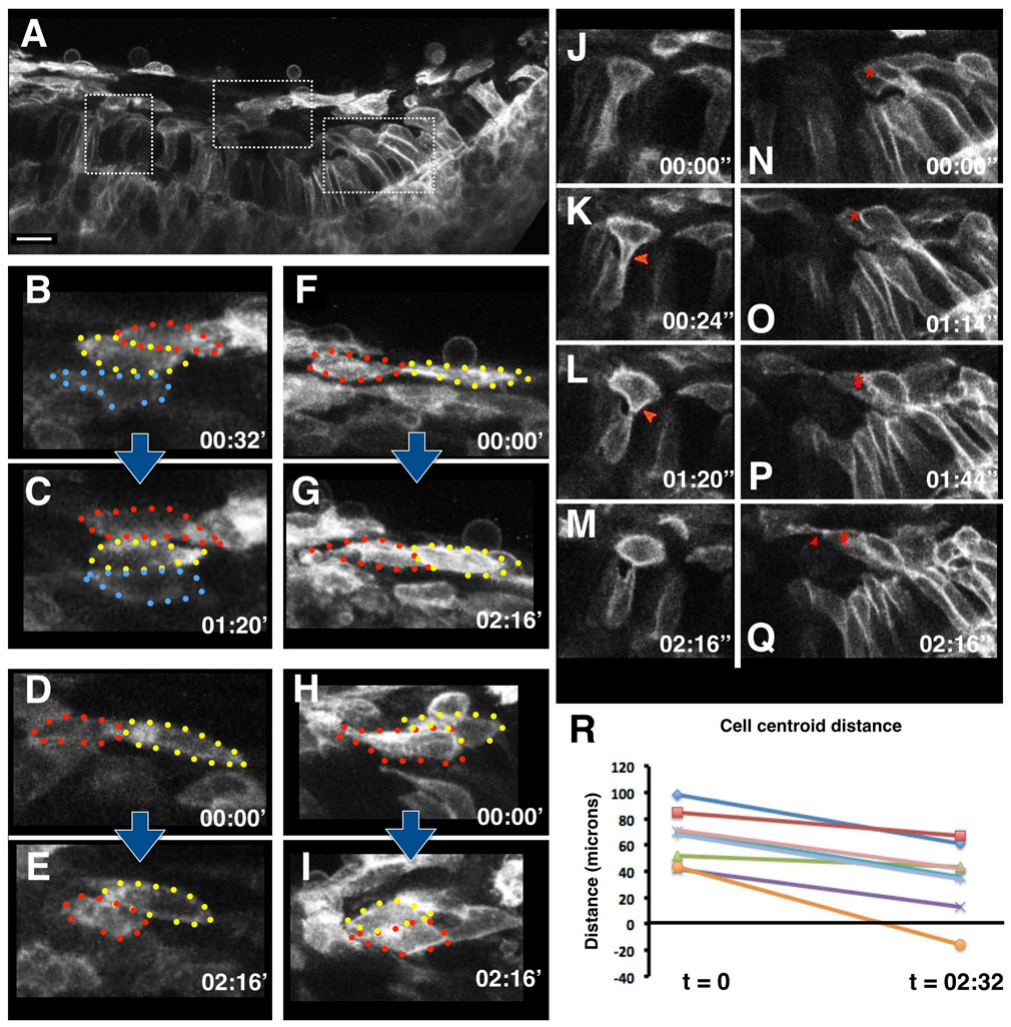
Suprabasal cells converge and extend via cell intercalation. **A.** Still from a confocal time-lapse video (S4 Movie) of a randomly GFP-labeled E12.5 tooth slice culture. **B,C.** Frames from the middle boxed region in (A) showing three individual cells rearranging themselves from an oblique to a vertical stack (see also S4 Movie inset). **D–I.** Three representative pairs of additional intercalating cells showing their initial (D,F,H) and final (E,G,I) positions at the indicated times (hours:minutes). Blue arrows between panels D–E, F–G, and H–I indicate paired stills. **J–Q.** A close-up of the left (J–M) and right (N–Q) boxed regions in panel A showing an example of a central delaminating cell (red arrowhead points at retracting basal contact (S6 Movie) and a lateral delaminating cell (marked with red asterisk) making contact (red arrowhead) with a suprabasal cell (see S6 Movie and text for details). **R.** Quantification of intercalation of cell pairs showing decrease in horizontal distance between their centroids during time-lapse period (t, shown in hours;minutes). Value below zero (yellow line) indicates a cell pair in which one cell moves right past another (corresponds to panels H,I). *n* = 8 from three separate explants.

In convergently extending tissues in other systems, contractile forces are produced by the active intercalation of elongated neighbouring cells between one another [5,19,21]. To observe dynamic cell behaviours in the tooth placode, we made confocal movies of tooth primordia under conditions that faithfully recapitulate normal tooth morphogenesis [22]. This confirmed that suprabasal cells converge toward the middle of the placode as it invaginates (S4 and S5 Movies), where they undergo active intercalation, sliding over one another at many suprabasal locations (Fig 2 and S4 Movie). Often, intercalating cells display highly dynamic, lamelliform protrusions (S4 Movie). We examined multiple labelled cell pairs in different specimens and observed widespread cell intercalation as revealed by the significantly changing planar distance between cell centroids (*p*-value < 0.025; *t* test; Fig 2R). This demonstrates that the convergence of the suprabasal cell tissue of the tooth is associated with cell intercalation. However, in sharp contrast to CE in Xenopus mesoderm [5], and the recently described CE in mouse prenatal eyelid closure [23], both of which are fibronectin (FN)-enriched and FN-dependent, we did not find detectable levels of FN in suprabasal or basal placode tissue (consistent with a number of published papers and databases), indicating that placode invagination is mechanistically distinct from these known instances of CE.

**Fig 3:**
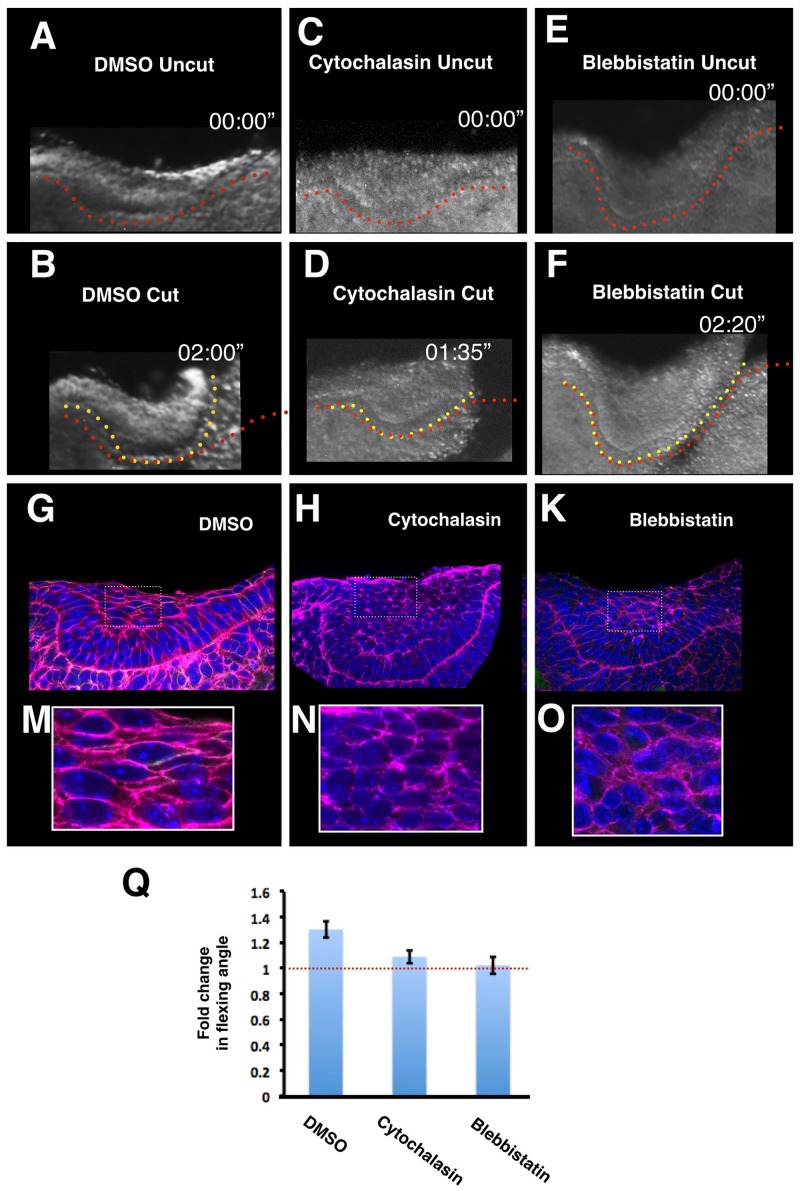
Tooth epithelium bending and suprabasal cell elongation depend on actin-myosin. **A–F.** Explants treated for 30 min with vehicle (medium plus DMSO) (A,B), the actin filament disruptor cytochalasin D (C,D) or the myosin contractility inhibitor blebbistatin (E,F) shown before cutting (A,C,E) or at the indicated times after cutting (B,D,F). Dotted lines show the position of the basal lamina before (red) and after (yellow) post-cutting incubation. **G–L**. Confocal images of fixed explants following 30-min treatment with vehicle or the indicated inhibitors showing that actin and myosin inhibition result in loss of cell and nucleus elongation. **M.** Quantification of inhibition shown in panels A–F. For each treatment *n* = 3. Values are mean +/- SD.

We next asked how this cellular behaviour of dynamic cell intercalation is able to generate force at the tissue level. To address this, and to further understand the molecular agents that generate force, we carried out a series of experiments using chemical inhibitors against components of the actinomyosin contractility machinery. Both an intact F-actin cytoskeleton and myosin contractility are required to generate lamelliform protrusions and dynamic cell motility observed in intercalating cells [24–26]. Brief culture of tooth slices in the presence of cytochalasin and blebbistatin inhibited bending upon lateral cutting (Fig 3A–3F and 3M). Such loss of tension across the suprabasal cells was accompanied by a loss of elongated cell shapes (Fig 3G–3L), commonly associated with cells engaged in force-producing intercalation movements [24]. This demonstrated that, upon inhibition of the molecular agents known to generate tension and elongated cell shapes in a subcellular level, a concomitant loss in tissue-wide tension is observed, thereby establishing a link between cell intercalation behaviour and tissue tension.

**Fig 4:**
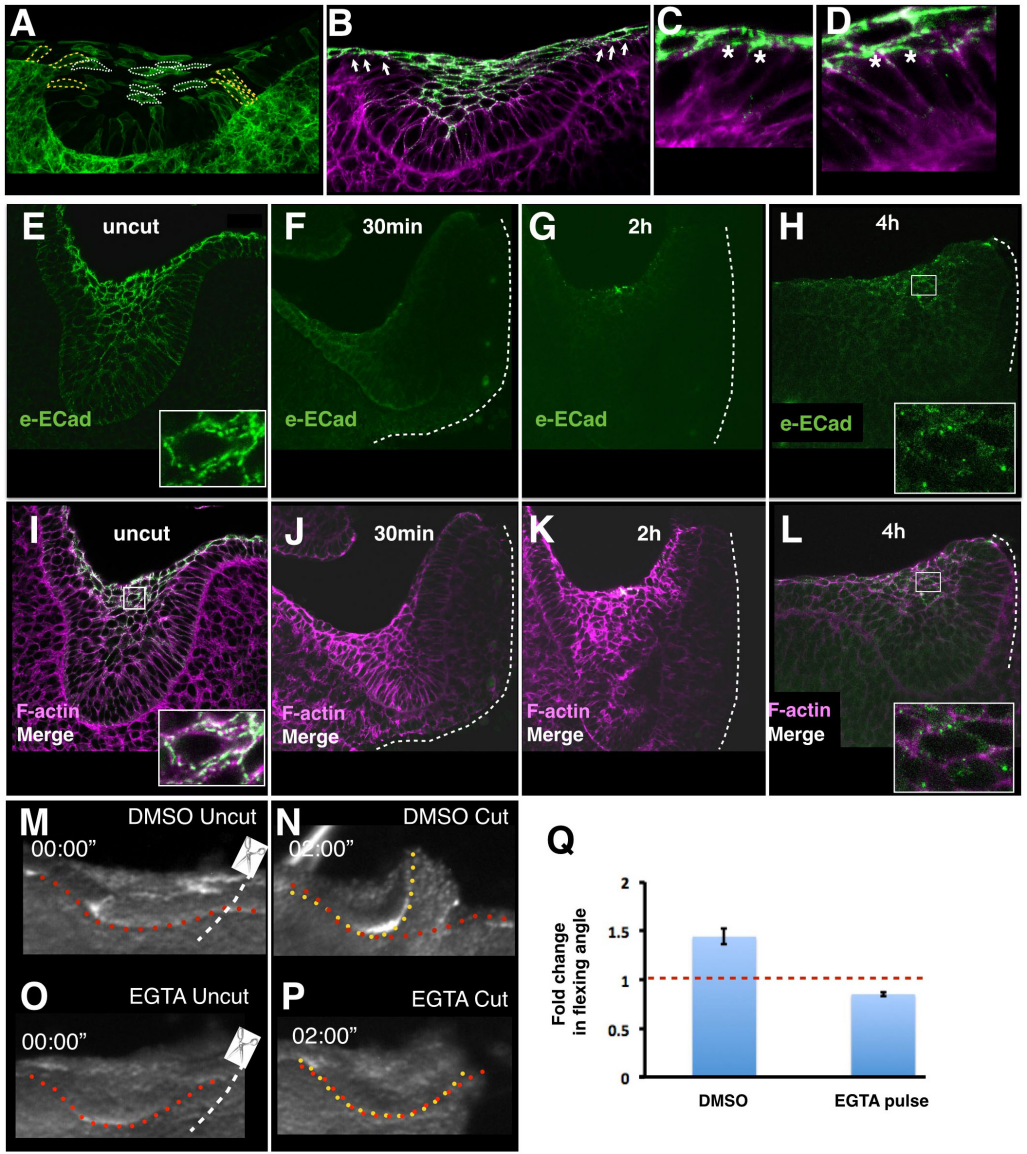
Suprabasal cadherin junctions depend on tissue-wide tension and calcium ions. **A.** Overview of mosaically stained tooth germ indicating location of elongated shoulder cells (yellow outlines) versus fully delaminated suprabasal cells (white outlines). **B–D.** Overview (B) and close-ups (C,D) of E12.5 tooth stained with anti-DECMA-1 (extracellular domain of E-cadherin, eECad, green) and phalloidin (magenta). Shoulder cells (marked by white arrows in B, asterisks in C,D) show punctate apical E-cadherin staining in apical protrusions **E,I.** Frontal view of an E12.75 tooth primordium stained with anti-E-cadherin (green channel) and phalloidin (B, magenta), showing staining in suprabasal cells. Detail (insets) show punctate pattern. **F–L**. Representative matched samples fixed 30 min (F,J), 2 h (G,K), and 4 h (H,L) after a lateral cut (marked by dashed white lines) showing loss and gradual re-emergence of E-cadherin signal. These are representative samples of *n* = 4 replicates for each time point and were stained side-by-side with the shown uncut controls. The image brightness was increased digitally for panels F–H to reveal background cadherin signal in intact tissue. **M–P**. Frames from timelapse videos of tooth germ explants incubated for 10 min with DMSO (panels M,N) or EGTA (panels O,P) before (M,O) and after (N,P) lateral cut showing inhibition of bending by the calcium chelator EGTA. **Q**. Quantificaiton of experiment shown in panels M–P.

We next asked if the cells at the tooth “shoulders” couple suprabasal contraction to the basal layer and analysed their shapes and behaviour. These were dramatically elongated cells that extended apical protrusions that leaned toward the midline of the invagination (outlined in yellow, Fig 4A). They also exhibited dynamic apical protrusions, which directly interacted with lamelliform protrusions of intercalating suprabasal cells (Fig 2N–2Q and S6 Movie) (unlike more central cells, which emerged apically in a rounder shape (Fig 2J–2M and S6 Movie). These apical protrusions appeared to contain E-cadherin (Fig 4B–4D), a normally suprabasal marker, despite the basal anchoring of the shoulder cells. These observations show that peripheral basal cells participate at their apical ends in the adhesive and intercalatory processes of suprabasal tissue. The existence of these cells reconciles the appearance of cells actively leaving the basal layer and yet still exerting a force on it: they are delaminating despite the traction of their basally-anchored neighbours, presumably pulling themselves up by their apical/centripetally-pointing ends while being pulled on by their basal ends.

Cadherin junctions are well known to transmit forces within epithelia [9,10,12,20], but linkage to tension in highly motile, suprabasal placodal tissue has never been examined. In vitro, the size of E-cadherin adherens junctions increases when tensile force is applied [27,28], implying that releasing tension should shrink junctions. Accordingly, we found that upon releasing tissue-wide tension by lateral cutting, suprabasal E-cadherin puncta (spot adherens junctions) detected by an antibody specific to the E-cadherin extracellular domain diminished to background levels within 30 min (Fig 4E, 4F, 4I and 4J), suggesting junction disassembly. Phalloidin staining was also reduced around the cut, indicating some local disassembly of actin filaments, but remained detectable, showing generally intact tissue. The suprabasal tissue remained intact and E-cadherin puncta reappeared after 2–4 h (Fig 4G, 4H, 4K and 4L), indicating that lateral cuts do not result in tissue death or dissociation. Assuming only that E-cadherin focally concentrated at cell interfaces—detected as we did using an antibody against the extracellular domain—is involved in cell adhesion, these findings together show the presence of a feedback loop between cell–cell adhesion and tissue-wide contractility in vivo. To begin to test whether E-cadherin adhesion might be required, we treated explants briefly (10 min) with EGTA, a calcium chelator that dissociates cadherin adhesions, which are calcium-dependent. This treatment did not lead to tissue dissociation but inhibited tissue recoil following a lateral cut (Fig 4M–4Q). This is consistent with an E-cadherin requirement, although it does not exclude other adhesive mechanisms.

**Fig 5:**
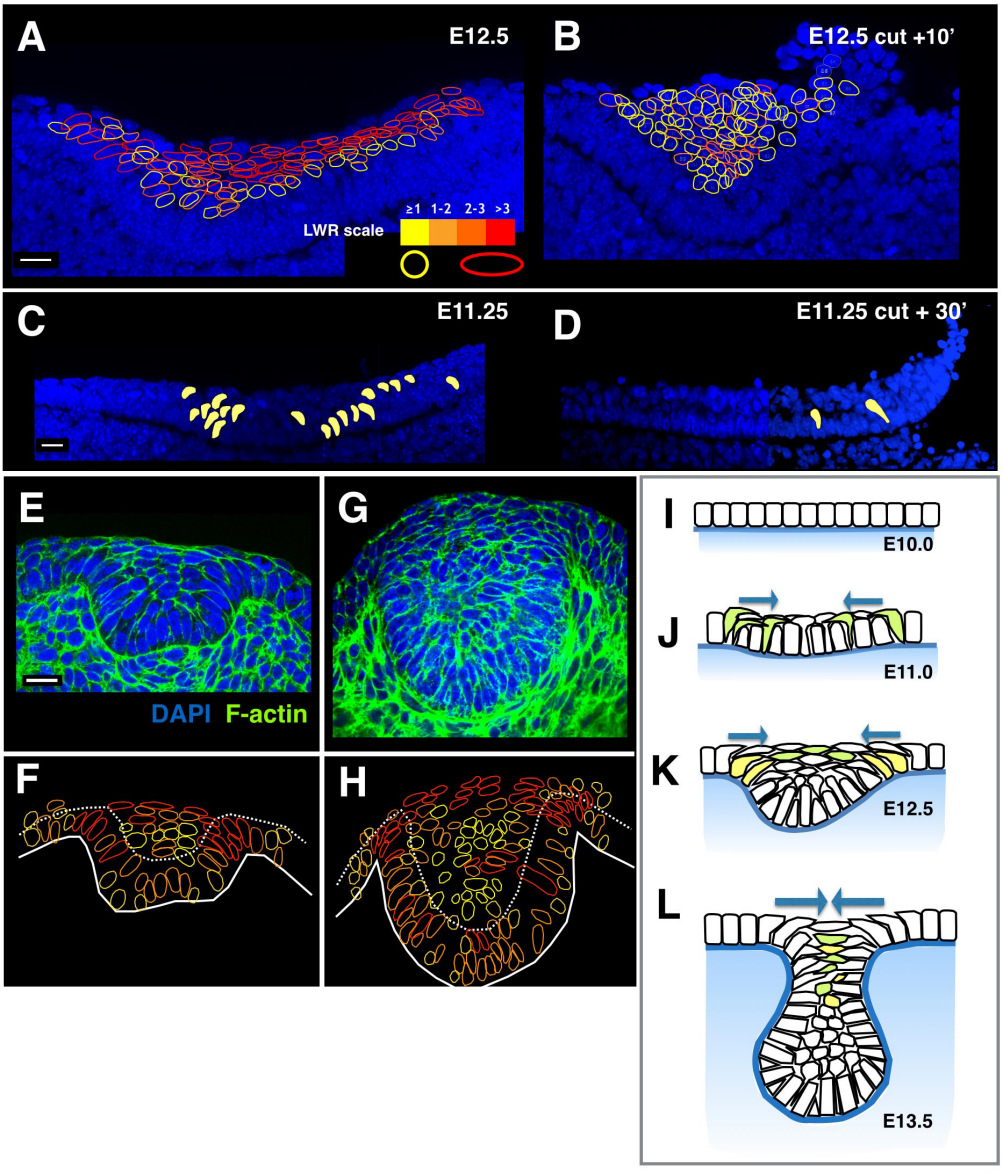
Nuclear shape signature reveals similar cell tensions in other placodes, and cell–cell traction is propagated through the tissue via tension-responsive cadherin junctions. **A.** Image stack projection of invaginating tooth placode explant showing individual nuclei outlined in colors indicating the length–width aspect ratio of their maximum size in the frontal plane, using the scale shown in the bottom right. **B.** The contralateral explant from the same embryo fixed shortly after a lateral cut. (Cut tissue is visible in the top right-hand corner of the panel, curling up and over as intact tissue, not cells or tissue layers being shed.) Suprabasal cell rounding is highlighted as an overall shift from predominantly red to yellow colour according to the scale in A. The experimental and control tooth slice cultures in A and B were dissected in the same medium and fixed at the same time. **C,D.** E11.25 tooth placodes before (C) and 30 min after (D) a lateral cut showing locations (projections from image stack) and disappearance of “banana” nuclei upon cutting. All scale bars are 30 μm. See also S3 Fig. **E,G.** Transverse views of an E13.5 hair follicle (E) and a mammary gland invaginating primordium (G). **F,H.** Outline drawings of cell nuclei from each micrograph are shown, colour-coded using the same aspect ratio scale as in panel A. **I–L.** Diagrammatic summary of our proposed model for ectodermal placode invagination. Cells of the cuboidal oral epithelium (I) become columnar and begin to delaminate apically and extend apical protrusions toward the centre of the structure (green cells in J), exerting tension suprabasally even before the tissue stratifies to create a shallow bend. As they delaminate and migrate centripetally, they form a canopy of elongated suprabasal cells that intercalate when they meet in the centre of the invagination, while more cells extend centripetally from the shoulders (yellow cells in K). By E13.5 the arc of elongated suprabasal cells have stacked on top of each other into a narrower-longer array that form the “neck” of the tooth bud (L). Periderm, a superficial layer of squamous cells present throughout the epithelium in tooth and non-tooth forming regions, has been omitted for clarity.

Nuclear deformation is another widely useable in vivo force sensor, because relaxed nuclei are spherical and require external forces to become ellipsoid [29,30]. Consistent with this, we observed that, in intact tooth placodes, nuclei of superficial cells had high aspect ratios in explants (Fig 5A) and in vivo in the direction of cell intercalation (but not in the orthogonal-*z*-axis; observe “lentil” cell shapes demonstrated in 3D in S7 Movie). Crucially, these shapes rapidly relaxed to a rounded shape upon tissue tension release following a lateral or suprabasal cut (Fig 5B). Consistent with this, cytochalasin and blebbistatin treatments that inhibited contractile tension also resulted in rounder nuclear shape (Fig 3G–3L). Interestingly, in 3D, the nuclei in vivo were lentoid (i.e., extended along the jaw as well as mediolaterally), consistent with centripetal tension around the whole placode (S6 Movie). Shoulder cells, too, showed tension-dependent elongation that was not only precisely correlated with invagination, but also relaxed upon lateral cutting, albeit more slowly than suprabasal cells (S3A–S3F Fig). At early stages of invagination (E11.5-E12.0) when any cells emerging from the basal layer to move toward the centre of the placode must “turn a corner,” we also observed a few, but striking, “banana”-shaped nuclei (Fig 5C), which relaxed within 30 min of a lateral cut (Fig 5D). This relaxation occurred on both sides of the invagination, indicating that tissue tension is propagated across the prospective tooth epithelium even before substantial placodal thickening has occurred. All the above nuclear deformation observations and functional relaxation experiments together demonstrate a direct link between cell-level tension and tissue-level contraction, thus mapping the forces involved.

The characteristic pattern of nuclear deformations allowed us to compare tooth primordia with forming hair follicles and mammary glands. Sectioning in the vertical plane revealed that all these invaginating structures contain the same patterns of nuclear elongation: arcs of cells with elongated nuclei linking suprabasal cells with inward-leaning basally attached shoulder cells (Fig 5E–5H). Cell tension mapping by nuclear deformation thus indicates that there is a tensile canopy of cells anchored at its periphery by a region of basal cells attached to the basal lamina in all these invaginating placodal structures. These findings suggest a new interpretation of the previously reported live imaging of centripetal cell migration in hair follicles [17]. Rather than migrating and converging to accumulate as a pile of cells, our analysis identifies these cells as likely being involved in active tension generation to converge placode shoulders, bending and buckling the underlying basal lamina (Fig 4E–4H).

## Discussion

Taken together, our findings demonstrate that tooth placode suprabasal cells use dynamic rearrangements to form a contractile structure, which is capable of driving invagination concomitantly with stratification. At early stages, the suprabasal contractile tissue bends the noncontractile basal layer in a gentle curve (Fig 5I and 5J) while later, as nonintercalating cells emerge centrally, the contractile tissue forms a canopy over the invagination to draw the shoulders (rim) into close approximation (groove or pore) (Fig 5K and 5L). The contractile structure driving invagination in the ectodermal placodes is topologically somewhat analogous to the contractile actin cables that drive invagination by apical constriction in epithelial monolayers, but on a vastly different scale: intercalation among cells rather than actin and myosin filaments. Finally, the similarity, right down to the distribution of force-deformed nuclei, between tooth bud, hair follicle, and mammary gland primordia indicates that these ectodermal organs share the same mechanisms of physical morphogenesis. It seems highly likely that similarly detailed inspection in other organs that form by invagination of stratified placodes may reveal a similar morphogenetic signature at the cellular level.

## Methods

### Mouse Strains and Genetic Labelling

All animal experiments were conducted under licence from the United Kingdom Home Office and the institutional Ethical Review Board. Tissue-cutting experiments were performed on wild-type CD1 mice. All cell shape analysis and live imaging was performed using mT/mG mice [8] as follows: Mosaic labelling was achieved by injecting 4 mg per 30 g body weight tamoxifen into a pregnant female mT/mG mouse [Gt(ROSA)26Sortm4(ACTB-tdTomato,-EGFP)Luo/J (Jackson Laboratories strain 007576)] crossed with a tamoxifen-inducible Cre male B6.Cg-Tg(CAG-cre/Esr1*)5Amc/J (Jackson Laboratories strain 004682).

### Tissue Processing

For all tissue cultures (live imaging and cutting experiments), tissues were cultured in phenol red-free Dulbecco’s Modified Eagle Medium: Nutrient Mixture F-12 (DMEM/F12).

For staining, E12.5 mouse heads were fixed in MEMFA (3.7% formaldehyde in 0.1 MOPS, 1 mM MgSO4, 2 mM EGTA) and manually cut, using a disposable microtome blade, into approximately 250 μm frontal slices and stained with Alexa Fluor 633 Phalloidin, DAPI (Molecular Probes), anti-phosphomyosin light chain antibodies (Cell Signaling Technology 3674) or anti-DECMA-1, an antibody against the ectodomain of E-cadherin (Sigma U3254). For live imaging, slices were cultured under coverslip fragments anchored at the edges with Vaseline and imaged in Lumox culture dishes (Starstedt).

### Imaging and Image Analysis

All confocal imaging was performed in a Leica SP5 inverted confocal laser scanning microscope using a 20X HCX-PLAPO dry objective lens, n.a.1.7. Z-stacks were acquired at half-optimal z. For cell shape analysis, z-stacks of individual cells were projected into a single plane in order to capture their maximum dimensions in xy. As the analysis was of bending as seen in the frontal plane, the posterior and anterior ends of the tooth primordium were excluded. In order to differentiate between short, intermediate, and tall cells, nuclear heights were measured (using the mid-height of the nucleus) and plotted as a proportion of each cell’s height and the basal palisade height.

Cell widths were measured at the base, the apex, and at 1/4, 1/2, and 3/4 of the cell’s height. As a measure of a cell’s shape with respect to contributing to epithelial bending, we wished to capture deformations of the cell body rather than protrusions at basal and apical ends. Therefore, base-to-apex ratios shown in Fig 1E and 1F are calculated using widths at 1/4 and 3/4 height. To partition the tooth epithelium in flat and concave, the radius of curvature of the basal lamina was measured using the three-point curve plugin in ImageJ, at a resolution of three cell widths.

For nuclear aspect ratio quantification, tissues were imaged in 3D and the section with the maximum size in the frontal plane for each nucleus was used to measure the major and minor axis lengths. Sections were taken only from the middle of the tissue in which elongation is strictly in the frontal plane. Absence of elongation in the *z*-axis after cutting was confirmed for nuclei in all specimens to rule out rotation.

### Cutting Experiments

For cutting experiments, we utilised the well-established slice culture system for studying tooth development ex vivo [31–34]. Briefly, E12.5 embryonic jaws (both maxillae and mandibles) were manually dissected from embryos in phenol-red-free Dulbecco’s Modified Eagle Medium: Nutrient Mixture F-12 (DMEM/F12) and placed on a sterilised tissue chopper stage (McIlwain tissue chopper, Ted Pella Inc.) on which 250 μm slices were cut. This corresponds to six to eight cell layers, optimal to include enough cell layers to preserve histomorphology (compared to fresh-fixed material) but thin enough to allow air access for extended development in vitro (as published [31–34], and routinely in our department for several days, although we never went beyond 30 h). Frontal slices were then transferred in polyHEMA (poly-hydroxyl-ethyl-methacrylate; Sigma)-treated tissue culture plates in culture media (see above) and immobilised using a fragment of coverslip held down with Corning vacuum grease. Cuts were performed using BD Micro-Fine insulin syringes and timelapse was recorded with minimal delay (capturing every 5 s) on a Nikon SMZ645 dissecting microscope with a MicroPublisher 5.0 RTV camera (QImaging). For lateral cuts in the presence of inhibitors, slices were cultured in 0.1 μg/mL cytochalasin D (C2168, Sigma) or 3 μg/mL blebbistatin (B0560, Sigma) for 30 min before performing the cut. Slices were fixed within 10 min of the cutting experiment and processed as above. Visual inspection of slice cultures after labelling with DAPI and fluorescently labelled phallloidin revealed that there were no obvious signs of cell death caused by the cut or culture conditions (S2G–S2I Fig).

### Cutting Experiment Quantification

The dimensions of the suprabasal cut width were measured from stills of the timelapse recording, immediately after the cut and at the end of recoil. The width of the cut at the oral surface was measured using the line tool and the command Analyse/Measure in ImageJ. To quantify tissue bending upon lateral, double cuts, and lateral cuts in the presence of cytochalasin and blebbistatin, the maximum angle of the basal lamina subtended at the centre of the invagination was measured using the ImageJ angle tool.

## Supporting Information

S1 Data Raw data for the plots shown in figures. (XLSX)

S1 Fig Ectodermal organ placode invagination and epithelial bending do not involve apical constriction. **A,B.** Existing models of cellular mechanisms for epithelial bending (invagination) are apical constriction with apically enriched contractile actomyosin (thickened black line) or basal wedging. **C.** A representative sample used for cell shape analysis; a 20 μm z-projection of confocal frontal slices, of an mT/mG;R26R-CreER E12.5 tooth with membrane GFP labeling and DAPI. Mosaic membrane GFP labelling outlined individual cells so that the ambiguity of assigning fluorescent signal between touching labeled neighbours was avoided, thus allowing accurate measurements to be made. Cell shapes were analysed in the basal cell layer (apical limit marked by white dotted line). White asterisks show examples of cells with basal nuclei, whose basal and apical widths were measured. *N* = 43 cells, which were measured from four different biological specimens. **D.** Proportional basal cell abundance in bent epithelium versus flanking flat epithelium, calculated as (number cell type)/(total number of cells) in respective region. **E.** Average cell basal and apical widths do not significantly differ in bent versus flat epithelium. **F.** Average cell base-to-apex width ratio shows a correlation of wedge-shaped cells with tissue invagination (note that the means of the ratios shown are not expected to be the same as the means of the averages shown in panel E). All error bars are +/- SDs. **G.** Phosphomyosin staining of an E12.5 tooth primordium (mesenchymal staining has been cropped out for clarity). **H,I.** Details of (G) showing that basal layer cells in the concave region have no apical phosphomyosin enrichment (H) or F-actin (I) enrichment.
(TIF)

S2 Fig Arc tension recoil is produced specifically from elongated suprabasal cells, with no contribution from basal layer cells. **A,B.** Stills from timelapse video recording (S3 Movie) of a cut of similarly thickened, non-invaginating nasal epithelial tissue cut showing no recoil. **C–F.** Tooth placode frontal slices before and after a lateral cut in the presence of cytochalasin D (E,E**′**) and blebbistatin (G, G**′**) (no flexion). **G–J.** Frontal tooth slice before and at the end of recoil produced by a suprabasal cell cut. **I,J.** Details of the boxed region in G and H, respectively. The dotted white lines mark the basal lamina, showing that, after indicated seconds, the basal lamina below the cut begins to kink (white arrowhead in J). **K.** Confocal Image of a frontal tooth slice fixed 2 min after performing a suprabasal cut experiment and stained with DAPI and AlexaFluor 488 Phalloidin. Only four of the most superficial suprabasal cell layers have been severed, leaving the basal palisade intact (thick white dashed line outlines the apical limit of the basal palisade and thin dotted line outlines the basal lamina). Areas of this image were manipulated only to achieve a more uniform brightness. Size bars in G and I are 30 μm, and size bar in K is 100 μm.
(TIF)

S3 Fig Shoulder nuclear shapes correlate with tissue curvature and tension. Shoulder cell nuclei were more apicobasally elongated than their non-shoulder neighbours and aligned with the direction of suprabasal cell intercalation because of their lean (arrows in A–E). Increased aspect ratio following shoulder “lifting” (curvature) starts on the buccal (cheek side) first at E12.5 (A,D) but then becomes symmetrical as the lingual shoulder lifts (D,E). This elongation is lost when the tissue-wide tension is released upon a lateral cut (dashed line in C), as indicated by loss of peaks in aspect ratio (F). Note that the latter relaxation is slower than that in suprabasal cells (2–4 h versus 10 min).
(TIF)

S1 Movie Explanted tooth epithelium bends autonomously (left), and manual re-extension and release of bent epithelium demonstrated tissue elasticity (right), related to Fig 1. The epithelium was microdissected from the mesenchyme using fine needles and cultured separately. Care was taken to explant the basal lamina intact. Timestamp is in mm:ss, 1 frame = 5 s.
(AVI)

S2 Movie (Left) Lateral cut of a frontal tooth primordium slice showing rapid bending, related to Fig 1K and 1L, and (right) double cut of a frontal tooth primordium slice showing no flexing, related to Fig 1O and 1P. Timestamp is in mm:ss, 1 frame = 5 s.
(AVI)

S3 Movie Suprabasal cut of a frontal tooth primordium slice showing recoil, related to Fig 1M and 1N, and superficial cut of a frontal slice of thick nasal epithelium showing no recoil, related to S2A and S2B Fig (right). Timestamp is in mm:ss, 1 frame = 5 s.
(AVI)

S4 Movie Overview of 3 h confocal timelapse of frontal slice of a molar tooth primordium at E12.5, related to Fig 2A (left), and example of tooth primordium suprabasal cells intercalating, related to Fig 2B and 2C (right). Cells are mosaically expressing membrane GFP (left; see Methods for details). Three cells show neighbour rearrangements such that they form a vertical stack. Intercalating cells such as these extend long and dynamic lateral protrusions (yellow arrow three-quarters through the movie). Timestamp is in hh:mm, 1 frame = 8 min. Buccal side is to the left.
(AVI)

S5 Movie Multiple suprabasal cell intercalations related to Fig 2D–2I in nearby optical sections. Neighbouring suprabasal cells are seen starting as lateral neighbours (arrows) and partially stacking on top of each other (cells outlined in yellow and red lines at the start, middle, and end of the movie. Timestamp is in hh:mm, 1 frame = 8 min. Buccal side is to the left.
(MOV)

S6 Movie Example of tooth epithelium tall cell delamination event near the centre of the invagination, related to Fig 2J–2M (left), and example of a leaning tall cell delamination event at the side (shoulder) region of the invaginating tooth primordium, related to Fig 2N–2Q. Red arrow appearing halfway through the left movie indicates the formation and retraction of the cellular basal process. Shoulder delaminating cell (right) extends a dynamic apical protrusion that contacts the lateral protrusion of a delaminated suprabasal cell (red arrow, three quarters) through the movie.
(AVI)

S7 Movie Rotating view of a 3D-rendered z-stack of DAPI labeled suprabasal cells showing lentoid nuclear shapes. Suprabasal cells and nuclei are only elongated in the direction of cell intercalation (parallel to the basal lamina) but are rounded in the anterioposterior direction.
(AVI)

## Abbreviations

CE: convergent extension
FN: fibronectin
