# Supplementary figures and images for: Invagination of Ectodermal Placodes Is Driven by Cell Intercalation-Mediated Contraction of the Suprabasal Tissue Canopy
Source: PLoS Biol. 2016 Mar 9;14(3):e1002405. doi: 10.1371/journal.pbio.1002405 (PMC4784948; doi:10.1371/journal.pbio.1002405)

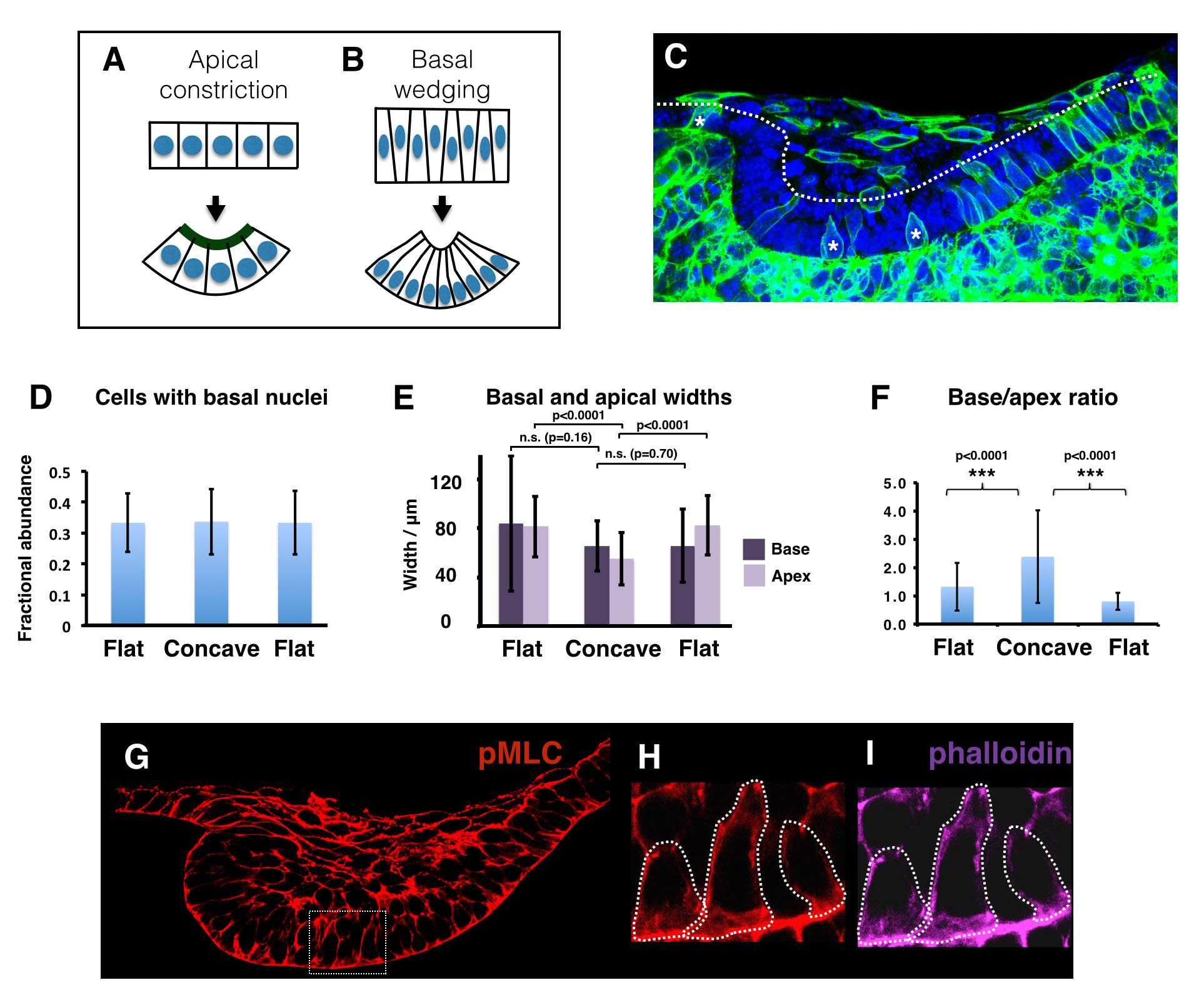

Supplement: S1 Fig — A,B. Existing models of cellular mechanisms for epithelial bending (invagination) are apical constriction with apically enriched contractile actomyosin (thickened black line) or basal wedging. C. A representative sample used for cell shape analysis; a 20 μm z-projection of confocal frontal slices, of an mT/mG;R26R-CreER E12.5 tooth with membrane GFP labeling and DAPI. Mosaic membrane GFP labelling outlined individual cells so that the ambiguity of assigning fluorescent signal between touching labeled neighbours was avoided, thus allowing accurate measurements to be made. Cell shapes were analysed in the basal cell layer (apical limit marked by white dotted line). White asterisks show examples of cells with basal nuclei, whose basal and apical widths were measured. N = 43 cells, which were measured from four different biological specimens. D. Proportional basal cell abundance in bent epithelium versus flanking flat epithelium, calculated as (number cell type)/(total number of cells) in respective region. E. Average cell basal and apical widths do not significantly differ in bent versus flat epithelium. F. Average cell base-to-apex width ratio shows a correlation of wedge-shaped cells with tissue invagination (note that the means of the ratios shown are not expected to be the same as the means of the averages shown in panel E). All error bars are +/- SDs. G. Phosphomyosin staining of an E12.5 tooth primordium (mesenchymal staining has been cropped out for clarity). H,I. Details of (G) showing that basal layer cells in the concave region have no apical phosphomyosin enrichment (H) or F-actin (I) enrichment. (TIF) [file pbio.1002405.s002.tif]

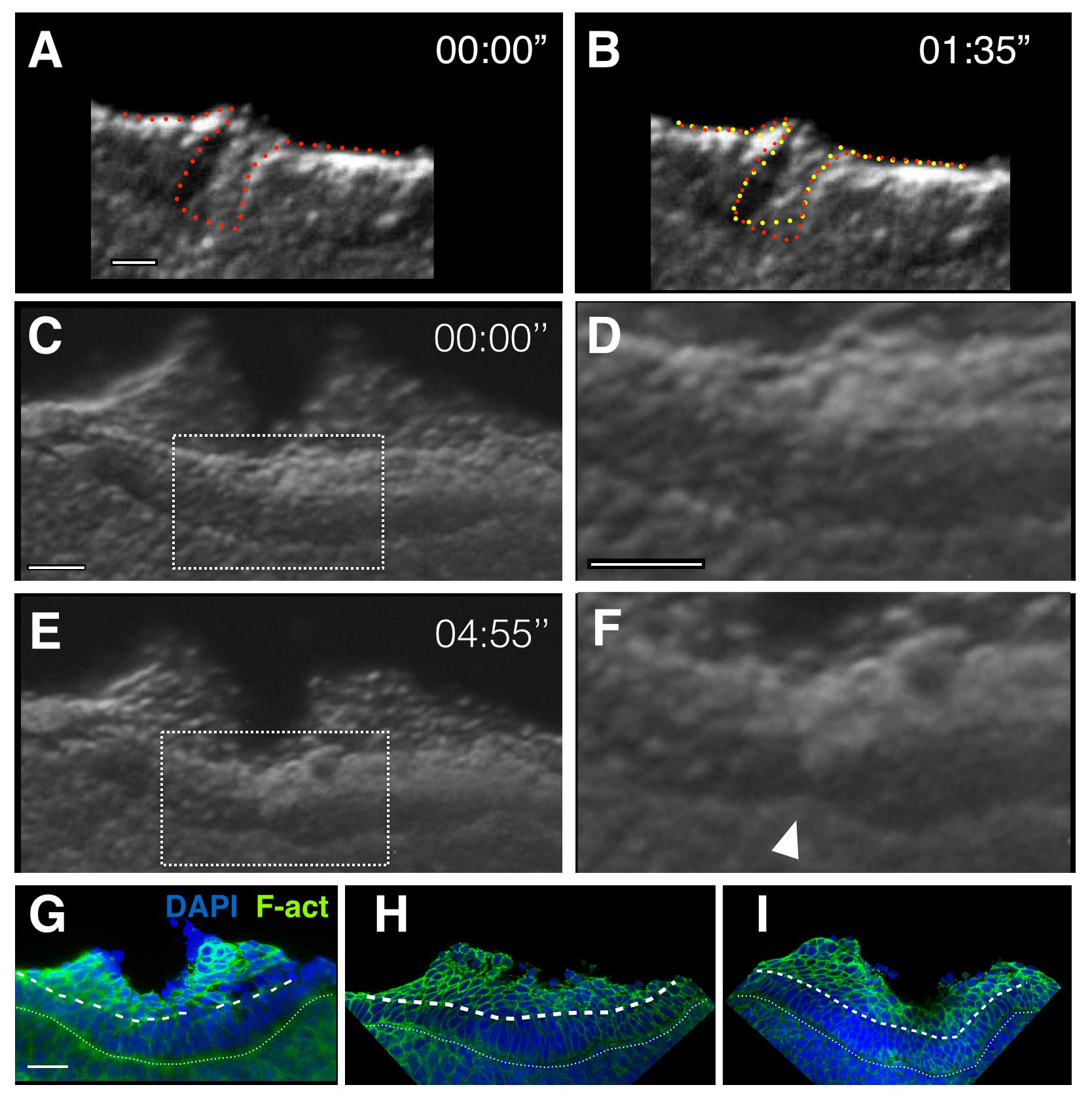

Supplement: S2 Fig — A,B. Stills from timelapse video recording (S3 Movie) of a cut of similarly thickened, non-invaginating nasal epithelial tissue cut showing no recoil. C–F. Tooth placode frontal slices before and after a lateral cut in the presence of cytochalasin D (E,E′) and blebbistatin (G, G′) (no flexion). G–J. Frontal tooth slice before and at the end of recoil produced by a suprabasal cell cut. I,J. Details of the boxed region in G and H, respectively. The dotted white lines mark the basal lamina, showing that, after indicated seconds, the basal lamina below the cut begins to kink (white arrowhead in J). K. Confocal Image of a frontal tooth slice fixed 2 min after performing a suprabasal cut experiment and stained with DAPI and AlexaFluor 488 Phalloidin. Only four of the most superficial suprabasal cell layers have been severed, leaving the basal palisade intact (thick white dashed line outlines the apical limit of the basal palisade and thin dotted line outlines the basal lamina). Areas of this image were manipulated only to achieve a more uniform brightness. Size bars in G and I are 30 μm, and size bar in K is 100 μm. (TIF) [file pbio.1002405.s003.tif]

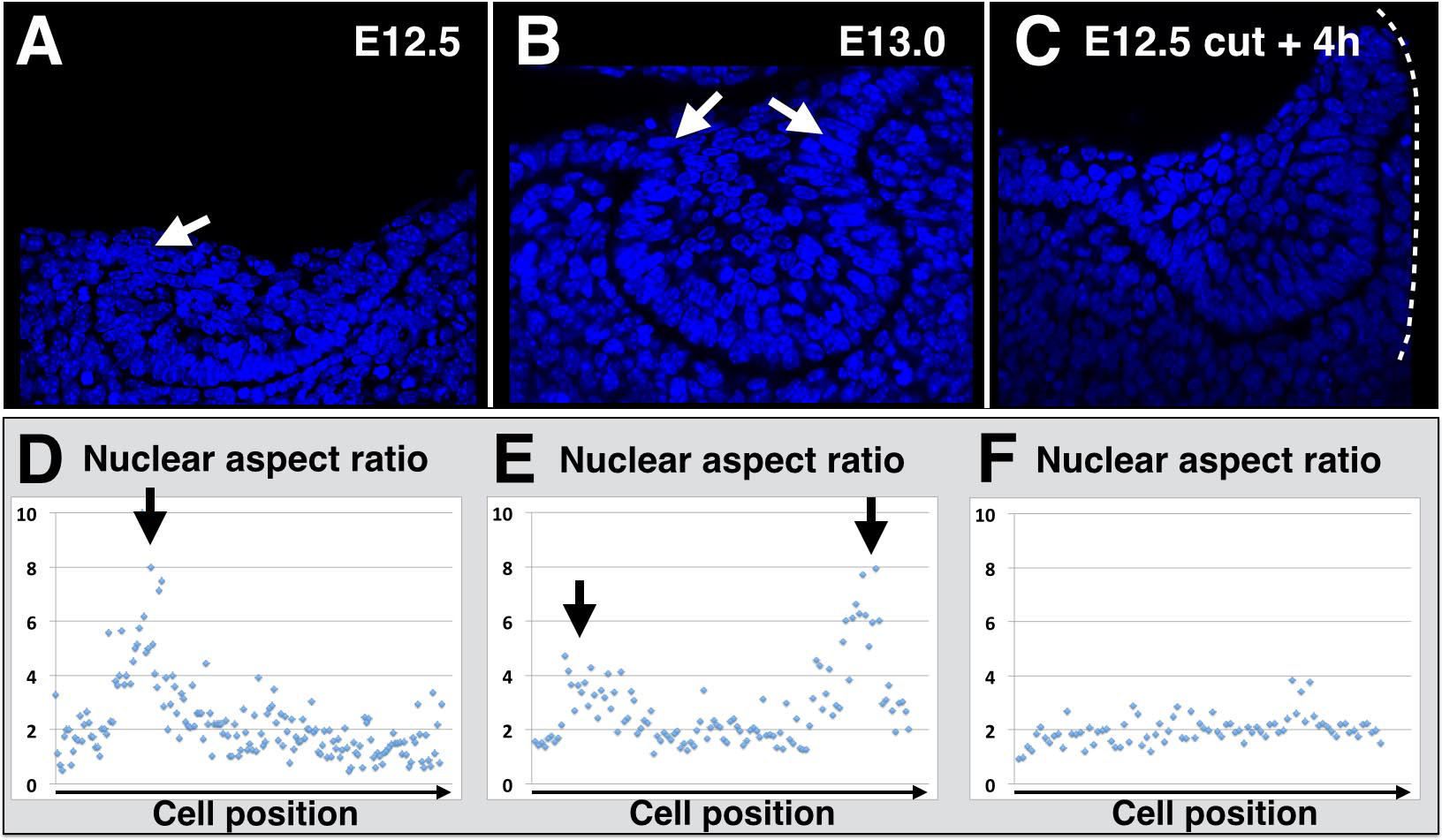

Supplement: S3 Fig — Shoulder cell nuclei were more apicobasally elongated than their non-shoulder neighbours and aligned with the direction of suprabasal cell intercalation because of their lean (arrows in A–E). Increased aspect ratio following shoulder “lifting” (curvature) starts on the buccal (cheek side) first at E12.5 (A,D) but then becomes symmetrical as the lingual shoulder lifts (D,E). This elongation is lost when the tissue-wide tension is released upon a lateral cut (dashed line in C), as indicated by loss of peaks in aspect ratio (F). Note that the latter relaxation is slower than that in suprabasal cells (2–4 h versus 10 min). (TIF) [file pbio.1002405.s004.tif]
